# The role of inflammation in silicosis

**DOI:** 10.3389/fphar.2024.1362509

**Published:** 2024-03-07

**Authors:** Tong-Tong Liu, Hai-Fei Sun, Yan-Xing Han, Yun Zhan, Jian-Dong Jiang

**Affiliations:** Institute of Materia Medica, Chinese Academy of Medical Sciences and Peking Union Medical College, Beijing, China

**Keywords:** silicosis, inflammation, immune cells, cytokines, signal pathway

## Abstract

Silicosis is a chronic illness marked by diffuse fibrosis in lung tissue resulting from continuous exposure to SiO_2_-rich dust in the workplace. The onset and progression of silicosis is a complicated and poorly understood pathological process involving numerous cells and molecules. However, silicosis poses a severe threat to public health in developing countries, where it is the most prevalent occupational disease. There is convincing evidence supporting that innate and adaptive immune cells, as well as their cytokines, play a significant role in the development of silicosis. In this review, we describe the roles of immune cells and cytokines in silicosis, and summarize current knowledge on several important inflammatory signaling pathways associated with the disease, aiming to provide novel targets and strategies for the treatment of silicosis-related inflammation.

## 1 Background

Silicosis is a progressive lung disease caused by long-term inhalation of silica particles. Previous studies elucidated that inflammation is associated with the pathogenesis and severity of silicosis. However, the associations between inflammation-related cells, factors, signaling pathways and proteins and disease have not been specifically studied and summarized in silicosis. In this review, we aimed to describe the role of inflammation during the development of silicosis and to explore the relationship between them, as well as to summarize current drug targeting on inflammation of silicosis.

## 2 Introduction

Silicosis is a fibrogranulomatous lung disease characterized by macrophage-dominated pulmonary alveolitis and restricted pulmonary function, which is traditionally been detected in miners but is now also being diagnosed in individuals working in contemporary industries such as denim production, domestic benchtop fabrication, and jewelry polishing (Leung et al., 2012; Barnes et al., 2019).

There are three types of silicosis—chronic, accelerated, and acute—which are categorized according to the quantity and length of silica exposure. Several additional diseases associated with silica exposure also result in considerable morbidity and mortality, including lung cancer, pulmonary tuberculosis, interstitial fibrosis, industrial bronchitis, hemoptysis, emphysema, rheumatoid complications, glomerulonephritis, and autoimmune disease, which negatively influence the quality of life and physical health of affected individuals (Rees and Murray, 2007; Pollard, 2016; Krefft et al., 2020; Faubry et al., 2021). Despite the high rates of silicosis incidence and mortality, treatment options for the prevention or repair of silicosis-associated lung damage are limited, and the pathomechanisms of the condition remain poorly understood (Li et al., 2022). Inflammation-related mechanisms are considered to underlie most cases of silica-induced lung injury. Elucidating silica-induced inflammation cascades and inflammation-fibrosis relationships is critical for understanding the pathophysiology of silicosis. In this review, we outline the most recent advances in the understanding of the inflammatory mechanisms associated with silicosis occurrence and development.

### 2.1 Silicosis and immune cells

Studies have demonstrated that silicosis begins with inflammation of the lungs. Silicon dioxide (SiO_2_) stimulates immune cells such as macrophages, neutrophils (NEUTs), mast cells (MCs), dendritic cells (DCs), T cells, and B cells. Immune cells regulate silicosis- and pulmonary fibrosis-related processes through several molecular pathways. Both the innate and adaptive immune systems are involved in the modulation of silicosis-induced inflammation (Langley et al., 2004; Malaviya et al., 2020).

#### 2.1.1 Macrophages

Macrophages are the most well-known immune cells in innate immunity during silicosis progression. Two kinds of macrophages, tissue-resident macrophages and recruited macrophages, play important regulatory roles in tissue repair and fibrosis. Alveolar macrophages (AMs) constitute a unique subset of tissue-resident macrophages with the highest content, which originate primarily from embryonic precursors during differentiation and development and self-renew during adulthood (Laskin et al., 2019). While recruited macrophages derived primarily from blood and bone marrow precursors become part of the lung macrophage pool during inflammation, they are referred to as recruited alveolar macrophages (RecAMs) (Aegerter et al., 2022). As demonstrated in an excellent review by Dang et al., AMs are mainly to detective and respond to microenvironment changes and phagocyte debris. RecAMs show a strong pro-inflammatory effect in inflammation and promoting fibrosis after inflammation regression (Dang et al., 2022).

**FIGURE 1:**
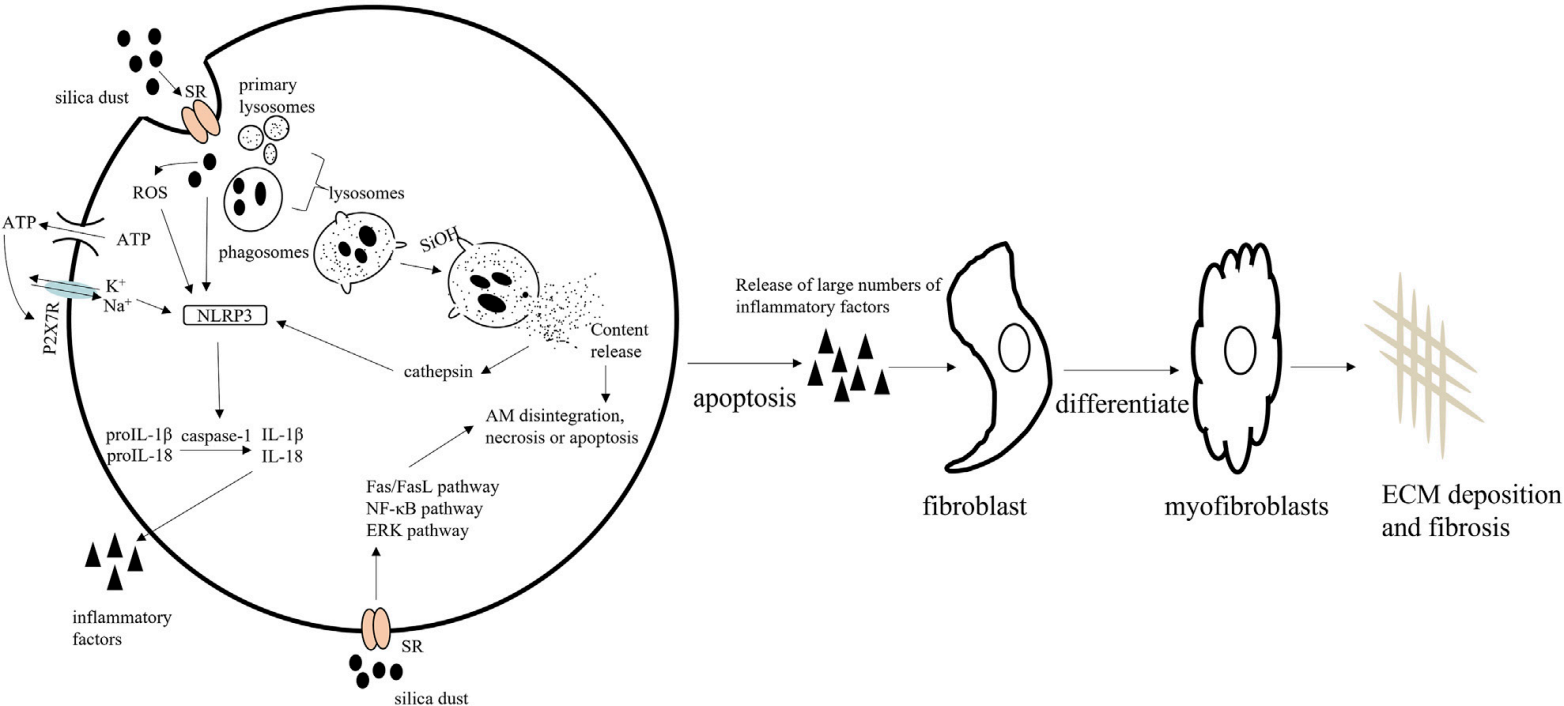
The mechanisms underlying the pathological effects of silica on alveolar macrophages (AMs).

AMs, also known as the resident innate immune cell of the lung, located on the luminal surface of the alveolar space, are the only macrophages exposed to air (Joshi et al., 2018). AMs represent the first line of defense against SiO_2_ and regulate the different stages of silicosis. When silica particles enter the lungs from the respiratory tract, scavenger receptors (SRs) on the surface of AMs first recognize and bind SiO_2_ particles (Lee et al., 2019). Subsequently, the SRs mediate the internalization of SiO_2_ via the formation of phagosomes, which then combine with primary lysosomes derived from the Golgi apparatus, yielding lysosomes, in which the phagocytic contents are degraded (Deretic, 2021). The integrity of the lysosomal membrane is disrupted throughout this degradation process via H-bonding reactions (SiOH interaction with oxygen and nitrogen groups on the membranes). Following lysosome rupture, the NOD-like receptor thermal protein domain associated protein 3 (NLRP3) inflammasome is activated by enzymes on the membrane, such as cathepsin, which triggers AM disintegration, necrotization, or apoptosis (Orlowski et al., 2015; Zhang et al., 2021). Damaged/dead AMs release a large number of inflammatory factors [interleukin (IL)-1β, IL-18, and others] and reactive oxygen species (ROS), thereby enhancing the pro-inflammatory cascade. They also stimulate a pro-fibrotic response that promotes the proliferation, activation, and migration of pulmonary fibroblasts. Fibroblasts synthesize and secrete collagen, and can also differentiate into myofibroblasts that release more extracellular matrix (ECM), leading eventually to fibrosis (Chanda et al., 2019), progressing to massive pulmonary fibrosis, and then to the impairment of respiratory functions (Hamilton et al., 2008; Todd et al., 2012). Subsequently, damaged AMs that are not eliminated continue accumulating and further activating the immune response. The SiO_2_ particles released by injured cells are ingested by new AMs, resulting in a vicious circle that serves to accelerate the fibrotic process (Tan and Chen, 2021) (Figure 1). These observations provide strong evidence that AMs are central to the pathology of silicosis in terms of both inflammation and fibrosis formation.

Studies have shown that silica engulfment results in lysosomal rupture, which leads to the accumulation of autophagosomes in AMs and the promotion of apoptosis via the mitochondrial apoptotic pathway (Tan and Chen, 2021). In addition, SiO_2_ mediates AM apoptosis through the Fas/FasL, NF-κB, and ERK signaling pathways. NF-κB and ERK activation exerts protective effects against silica-induced apoptosis in macrophages, whereas Fas/FasL promote pro-apoptotic effects in these cells (Gambelli et al., 2004; Yao et al., 2013).

Thus, altering the autophagy and apoptosis of AMs can reduce the silica-inflammation, which implies that manipulating macrophage autophagy may be a promising treatment target for pulmonary fibrosis. Dioscin and trehalose protecting the autophagy-lysosomal system leads to alleviate CS-induced apoptosis and cytokine production in AMs, which may provide concrete molecular mechanism for the therapy of silicosis (Du et al., 2019; Tan et al., 2020). Notably, atractylenolide III alleviates the apoptosis of AMs but inhibit autophagy by mTOR-dependent manner, thereby improving the blockage of autophagic degradation in AMs (Chen et al., 2021).

The number of macrophages in the lung increased in mice after exposure to silica (Song et al., 2022). Once localized at the site of lung injury, these macrophages are activated by mediators they encounter in the lung microenvironment and develop into subpopulations with varying degrees of pro-inflammatory (M1) or wound-repair (M2) activity (Atri et al., 2018). In the early stages of silicosis, M1 macrophages are stimulated, which promotes inflammation primarily through the production of the pro-inflammatory cytokines IL-1β and IL-6. Later, M2 macrophages are induced to secrete the pro-fibrotic cytokines IL-10 and TGF-β, which promotes tissue repair (Zhang et al., 2021; Fan et al., 2022; Tang et al., 2022). Meanwhile, exosome secretion is increased in macrophages treated with SiO_2_, which stimulates crosstalk between macrophages and fibroblasts. Additionally, the inhibition of endoplasmic reticulum stress reduces exosome secretion, which, in turn, suppresses myofibroblast differentiation, proliferation, and migration (Qin et al., 2021). Therefore, inhibiting macrophage polarization can reduce the symptoms of pulmonary fibrosis. Pirfenidone and bicyclol ameliorates pulmonary inflammation and fibrosis in a rat silicosis model by inhibiting macrophage polarization (Zhan et al., 2021; Tang et al., 2022).

AMs engulf invading silica dust. The lysosomal membrane of AMs is disrupted by H-bonding reactions, resulting in AM apoptosis. Simultaneously, silica dust induces NLRP3 inflammasome activation in AMs. Apoptotic AMs secrete large numbers of inflammatory factors. Eventually, fibroblasts proliferate and are activated and produce large amounts of extracellular matrix (ECM), leading to fibrosis. Macrophage apoptosis is regulated by the Fas, NF-κB, and ERK signaling pathways.

#### 2.1.2 Neutrophils

NEUTs play a pivotal role in the innate immune defense against pathogens, killing them directly by phagocytosis and degranulation (Khan et al., 2019). In response to silica insult, neutrophils secrete a wide range of cytokines as well as neutrophil extracellular traps (NETs), which stimulate the production of downstream factors and lead to the recruitment of more neutrophils or other leukocytes to the lung (Khan et al., 2019). The lungs of mice show severe injury after the inhalation of SiO_2_ for 28 days, and the content of NETs in the bronchoalveolar lavage fluid is significantly increased (Liu et al., 2019). Brinkmann et al. described extracellular DNA release in human neutrophils challenged with different doses of silica particles, suggesting that silica crystal-promoted NETs could play an important role in the establishment of silicosis (Brinkmann et al., 2012). Furthermore, it was reported that NETs induce the activation of pulmonary fibroblasts and their differentiation into myofibroblasts, leading to increased collagen production and fibrosis (Chrysanthopoulou et al., 2014). Airway neutrophils seem to be activated in silicosis, as reflected by the observed increase in their main proteolytic product, neutrophil elastase (NE), in the BALF of affected patients (Scharfman et al., 1989). Gregory et al. demonstrated that fibroblast and myofibroblast accumulation was significantly reduced in *Ne* ^*−*/*−*^ mice, which are protected from asbestos-induced pulmonary fibrosis. They further found that NE directly promotes lung fibroblast proliferation and myofibroblast differentiation (Gregory et al., 2015).

#### 2.1.3 Mast cells

MCs form part of the sentinel immune cell population. Despite the evidence supporting a role for MCs in silicosis, no relevant systematic studies on the effects of silica on these cells exist to date. Clinical studies have shown that MCs are activated in silica-induced inflammation. Increased numbers of tryptase- and basic fibroblast growth factor (bFGF)-positive MCs were found within silicotic nodules in lung tissues of patients with silicosis (Hamada et al., 2000). MCs not only recruit and activate other immune cells by secreting inflammatory mediators but also regulate vascular permeability, smooth muscle cell contraction, and fibroblast growth in lung fibrosis (Wygrecka et al., 2013). Jared et al. found that, unlike wild-type C57BL/6 mice, MC-deficient mice do not develop inflammation nor do they display significant collagen deposition following silica instillation (Brown et al., 2007). MCs also secrete tryptase, which interacts with multiple regulatory factors, such as activating proteinase-activated receptor 2 (PAR2), inducing DNA synthesis in resting fibroblasts and promoting collagen synthesis, thereby stimulating fibroblast proliferation (Henriques Á et al., 2016). Collectively, these studies highlighted a clear involvement of MCs in silicosis. Yiling et al. proposed the IgE-FcεRI axis as a molecular mechanism responsible for the activation of mast cells. They found that silica exposure induced MCs degranulation, as shown by the increased serum histamine levels and β-hexosaminidase activity in WT mice, but in FcεRI-deficient mice, no significant alterations in serum, indicating that MCs degranulation took place in silica-exposed mice, but was blocked by FcεRI deficiency (Chen et al., 2022). Chymase, chymotrypsin-like serine protease, is present in MCs, which can promote inflammatory responses. The specific chymase inhibitor TY-51469 suppresses the accumulation of MCs and NEUTs in the lung and reduces pulmonary fibrosis in silicotic mice (Takato et al., 2011).

#### 2.1.4 Dendritic cells

DCs are potent antigen-presenting cells (APCs) that modulate immune response initiation. DCs, recently identified players in the pathogenesis of silicosis, have been shown to accumulate in the lung tissue of silica dust-exposed rats (Bao et al., 2018; Liu et al., 2019). DCs migrate from the alveoli into the lung parenchyma in response to silica, resulting in significantly increased numbers of activated T lymphocytes, and thereby promoting immune activation (Beamer and Holian, 2007). Suna Liu et al. showed that the phenotype, function, and migration of DCs and the balance between T-helper1 (Th1) and Th2 cells are altered by silica exposure and that these changes contribute to the development of silicosis (Liu et al., 2019). Moreover, Lei Bao et al. demonstrated that DCs regulate the polarization of Th1/Th2 cells via CD80, CD86, MHC-II, and IL-12 expression (Bao et al., 2018). Combined, these observations indicate that DCs may play a critical role in modulating immune homeostasis during silicosis.

#### 2.1.5 Adaptive immune cells

It is known that adaptive immune cells infiltrate the silicotic lung. However, the precise role of different immune cell subsets in the pathology of silicosis has not been elaborated. In mice, SiO_2_ treatment was found to activate T and B cell proliferation via T-cell (TCR) and B-cell (BCR) receptor complexes and significantly alter the proportions and subtypes of T and B cells (Eleftheriadis et al., 2019; Zhao et al., 2022). The expression of PD-1/PD-L1 and CTLA-4 was also dysregulated in T and B cells. Additionally, although both PD-1/PD-L1 and CTLA-4 inhibitors improved silica-induced immune system disruption, only PD-1/PD-L1 signaling inhibition exerted significant ameliorative effects against silicosis (Zhao et al., 2022). Meanwhile, analysis of patients with this condition indicated that the morbidity of autoimmune diseases increased after dust exposure (Pollard, 2016).

Once silica dust has entered the respiratory tract, APCs, such as DCs, activate naïve T lymphocytes via the processing and presentation of silica antigens. CD4^+^ T cells, primarily Th1, Th2, Th17, and regulatory T cells (Tregs), have been demonstrated to exert pathogenic effects in silica particle-induced pulmonary fibrosis.

Th1/Th2 imbalance plays a regulatory role in the inflammatory phase of silicosis. The Th cell balance shifts from Th1 dominance during inflammation to Th2 dominance in the development of fibrosis. Specifically, IFN-γ and IL-12 produced by Th1 cells inhibit fibroblast proliferation and fibrous tissue formation, whereas Th2-secreted IL-4, IL-5, and IL-13 are thought to promote fibrosis by inducing fibroblast aggregation and activation (Song et al., 2014; Malaviya et al., 2020).

Th17 cells also play a key regulatory role during inflammation and fibrosis of the lung in response to silica exposure. IL-17, a signature cytokine of Th17 cells, contributes to lung inflammation and fibrosis by increasing IL-6, IL-8, and matrix metalloproteinases (MMPs) production (Lo Re et al., 2010; Kolahian et al., 2016). The Th17 response is key to promoting silicosis inflammation and fibrosis by affecting the homeostasis of Th cell-mediated immune responses and increasing the production of IL-22 and IL-1β (Song et al., 2014).

Tregs have dual functions, depending on the stage of silicosis and interaction with other immune cells. In one study, mice exposed to silica were administered anti-CD25 monoclonal antibodies to deplete Tregs. In the early inflammatory stage, Treg-depleted mice were found to be susceptible to severe inflammation, resulting in enhanced inflammatory cell infiltration and the promotion of fibrosis. In the later fibrotic stage, the same mice show a delay in silicosis progression (Liu et al., 2010). This suggests that Tregs play a detrimental role in lung fibrosis in the early stages of the disease, increasing the production of TGF-β and collagen deposition. However, the opposite is seen in the later stages (Boveda-Ruiz et al., 2013). In silica-induced fibrosis, Tregs promote Th17 cell differentiation and IL-17 secretion by regulating TGF-β and IL-1β and also interact with Th1/Th2 cells, altering Th1/Th2 polarization toward a Th2-dominant response by suppressing Th1 responses (Liu et al., 2010; Song et al., 2012).

B cells are also actively involved in silicosis. Regulatory B cells (Bregs) have immunomodulatory properties and secrete inhibitory cytokines such as IL-10, as well as fibrotic cytokines such as TGF-β (Rosser and Mauri, 2015). There is clear evidence that IL-10-producing Bregs (B10) are involved in the development of silicosis through the regulation of the Th cell balance. B10 deficiency results in an increase in the number of inflammatory cells (early lymphocytes, late macrophages, and neutrophils) and factors (IL-6 and TNF-α), which aggravates inflammation. Additionally, B10 insufficiency leads to a decrease in silica-induced TGF-β expression, which attenuates the pro-fibrotic response (Liu et al., 2016). The role of B10 in silicosis has also been confirmed in patients. The levels of IL-10 and Bregs are increased in patients with silicosis, resulting in Treg maintenance and the regulation of the Th1/Th2 immune balance (Chen et al., 2017). Kazuhiro Komura et al. found that a lack of CD19 weakens the B cell response and significantly reduces susceptibility to pulmonary fibrosis. In contrast, fibrosis is aggravated in mice overexpressing CD19, indicating that B cells play a pro-fibrotic role in pulmonary fibrosis. The authors proposed that B cells regulate cytokine expression, thereby promoting fibrosis via a hyaluronic acid-TLR4-dependent pathway (Komura et al., 2008).

**FIGURE 2:**
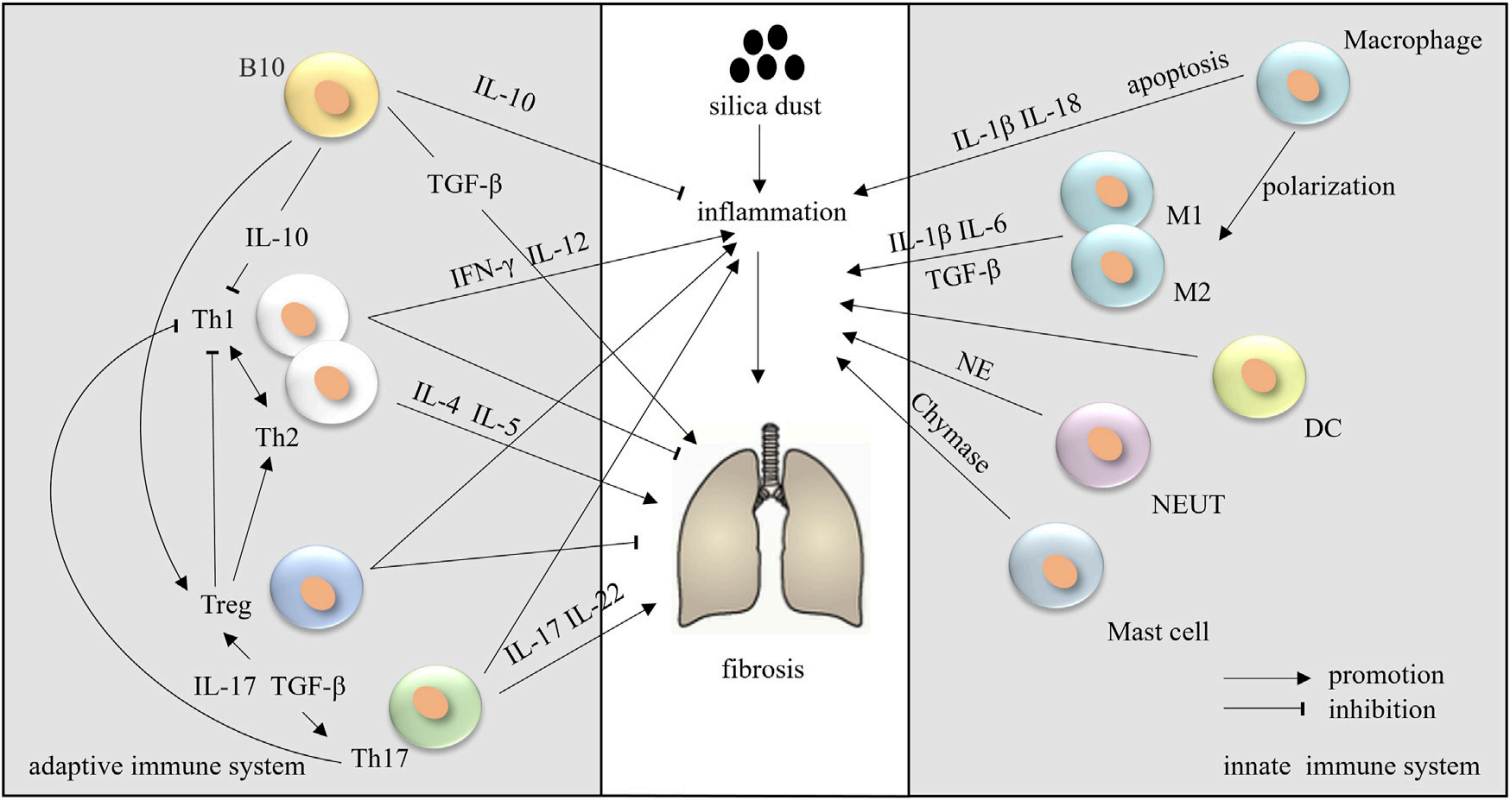
The innate and adaptive immune systems in silicosis.

During the development of silicosis, many immune cells are activated and produce multiple mediators that either activate or suppress inflammation and fibrosis (Figure 2).

## 3 Inflammatory factors

Pro-inflammatory cytokines released during inflammation by macrophages and, potentially, also neutrophils, MCs, and lymphocytes, promote the exaggerated development of silicosis. Cytokines that have received substantial attention include IL-6, IL-1β, TGF-β, TNF-α, and platelet derived growth factor (PDGF) (Blanco-Perez et al., 2021). The complicated interaction between these mediators will be elaborated in the following sections.

### 3.1 Interleukins

IL-1β is a downstream product of NLRP3 inflammasome activation and has pro-inflammatory and fibrinogenic effects in silicosis. The excessive secretion of IL-1β can aggravate silicosis, whereas its neutralization or knockout can reverse the progression of the disease (Cavalli et al., 2015; Qin et al., 2021). Studies have shown an association between *IL1B* gene polymorphism and an increased risk of silicosis (Weng et al., 2015). The importance of IL-1β in silicosis has been demonstrated in both the inflammatory and fibrotic stages. IL-1β can promote inflammatory cell infiltration by stimulating the expression of TNF-α, IL-6, and MCP-1, thereby further amplifying the inflammatory response (Guo et al., 2013; Chen et al., 2014). Moreover, IL-1β contributes to a shift in the Th1/Th2 balance toward a Th2-dominant response during the development of fibrosis (Guo et al., 2013). IL-1β is a pro-fibrogenic cytokine that enhances collagen deposition by increasing the expression of TGF-β (Fan et al., 2001; Guo et al., 2013).

IL-6 is mainly secreted by monocytes, lymphocytes, pulmonary fibroblasts, pulmonary macrophages, and endothelial cells. The levels of IL-6 are significantly increased in the serum and BALF of patients with silicosis (Blanco-Perez et al., 2021). High IL-6 levels exert marked effects on other inflammatory factors, immune cells, and oxidative stress, which are closely related to the pathogenesis of silica-induced pulmonary fibrosis. IL-6 is required for the production of other inflammatory factors, such as IL-1 and TNF-α. The differentiation of CD4^+^ T cells is also modulated by IL-6, which induces the differentiation of Th17 cells but inhibits the production of Tregs. An imbalanced Th17 cell/Treg ratio results in the disruption of the immune response and the promotion of inflammation-related diseases (Kang et al., 2019). In mice, silencing IL-6 receptor-α can ameliorate the secretion of Th2 cytokines (IL-4 and IL-5) and inhibit the activation of the IL-6/JAK/STAT pathway, thereby improving lung function and alleviating the development of silicosis (Tripathi et al., 2010). IL-6 can also influence dust-induced oxidative stress in the lung. The epithelial cells of the lungs produce ROS and express ICAM-1 through an IL-6/AKT/STAT3/NF-κB-dependent pathway, thereby mediating immune cell adherence and infiltration into inflammatory sites and fibrotic lesions (Liu et al., 2018).

IL-17, secreted by macrophages and Th17 lymphocytes, is important for host defenses against extracellular pathogens, and can exacerbate inflammatory responses (Korn et al., 2009). Rats exposed to silica display increased levels of IL-17, which is associated with the progression of fibrosis (Lo Re et al., 2010). The mRNA expression levels of IL-17, retinoid-related orphan nuclear receptor γt (RORγt), and IL-23 are increased in AMs of mice tracheally perfused with silica dust, leading to the local aggregation of Th17 lymphocytes and the production of IL-17. These changes subsequently aggravate the acute inflammatory response in silicosis (Lo Re et al., 2010). IL-23 maintains the phenotype of Th17 lymphocytes, while RORγt is the key transcription factor of Th17 lymphocytes (Korn et al., 2009). The neutralization of IL-17 can reduce neutrophil recruitment and suppress Th17 cell development by decreasing IL-6 and IL-1β content (Hasan et al., 2013). IL-17 modulates the differentiation of Tregs in the early phase of silicosis and promotes the Th1/Th2 immune response, thus influencing silica-induced lung inflammation and fibrosis by regulating the production of IL-22 and IL-1β (Chen et al., 2014; Song et al., 2014). Hence, neutralization of IL-17 with anti-IL-17 antibody could inhibit silica-induced lung inflammation and fibrosis by decreasing Th17 cells and IL-1β, increasing Tregs, and delaying silica-induced Th1/Th2 immune response (Chen et al., 2014).

Other interleukins, such as IL-13, IL-18, and IL-8, also promote fibrosis through a variety of mechanisms, while cytokines such as IL-7, IL-12, and IL-22 can inhibit the formation of fibrosis by reducing collagen deposition (She et al., 2021).

### 3.2 TNF-α

TNF-α, produced by activated macrophages, fibroblasts, and lymphocytes, can initiate a complex cascade of inflammatory responses in the early inflammatory stage and during the late development of silica-induced fibrosis (Park et al., 2012). TNF-α increases the risk of pulmonary fibrosis by inducing the expression of IL-1, IL-6, and IL-8. Meanwhile, TNF-α can stimulate the expression of TGF-β1 through the ERK signaling pathway (Xie et al., 2021), which promotes fibrosis progression by inducing ECM synthesis and deposition in fibroblasts (Morrison and Davey, 2009; Jiang et al., 2015). That TNF-α plays an important role in the development of silicosis has been demonstrated in SiO_2_-treated rats. After a course of infliximab (anti-TNF-α antibody) injections, the number of inflammatory cells was decreased, collagen deposition was reduced, NF-κB signaling was inhibited, and iNOS expression was downregulated, leading eventually to an improvement in pulmonary function (Zhang et al., 2018). Silicosis can be prevented with the administration of an anti-TNF antibody but is aggravated by exogenous recombinant TNF-α application. The latter augments fibroblast growth, collagen deposition, and cell necrosis (Piguet, 1990). Moreover, in silicosis, TNF-α stimulation promotes the secretion of other cytokines, influences the function of signaling pathways, and potentiates the proliferation of fibroblasts and the deposition of collagen. Infliximab is the anti-TNF-α antibody, which improve silica-induced pulmonary inflammation by decreasing the TNF-α, inhibiting NF-κB signaling as well as oxidant status (iNOS) (Zhang et al., 2018).

### 3.3 TGF-β

TGF-β is a well-characterized fibrogenic mediator and it has long been known that TGF-β expression is increased in silicosis. In rats, after a single intratracheal infusion of silica, TGF-β peptide was detected in fibroblasts and macrophages surrounding silicotic granuloma and fibroblasts adjacent to hyperplastic type II cells in the lungs (Williams et al., 1993). The major role of TGF-β in silicosis is the promotion of ECM accumulation, EMT induction, and the inhibition of matrix degradation. During pulmonary fibrosis, the TGF-β/Smad signaling pathway is activated, which increases the expression of collagen and fibronectin genes. TGF-β binds to TGF-β receptors (TβR) on lung fibroblasts, leading to the phosphorylation of Smad2/3. Phosphorylated Smad2/3 then translocate to the nucleus and induce the expression of genes that promote EMT and the transformation of fibroblasts into myofibroblasts, resulting in the production of a large amount of ECM (Attisano and Wrana, 2002; Zhang et al., 2021). An uncontrolled, continued EMT may result in fibrosis that has a close relationship with silicosis (Stone et al., 2016). TGF-β can also inhibit matrix degradation by decreasing the secretion of proteases and increasing that of protease inhibitors such as MMPs and tissue inhibitor of metal protease (TIMP) (Yang et al., 2011). Through these various means, TGF-β can cause pulmonary fibrosis by promoting the accumulation of a large amount of ECM in the lungs. TGF-β can also promote pulmonary fibrosis *via* the activation of the ERK and PI3K/AKT pathways (Kolahian et al., 2016). Hence, as a key inflammatory factor in silicosis, any way to downregulate the expression of TGF-β or to neutralize secreted TGF-β might be possible to delay the progression of silicosis. According to this, the anti-TGF-β antibody HTPEP-001 was developed and is currently in clinical phase I (Liu et al., 2023).

### 3.4 Growth factors

Many growth factors, such as PDGF, fibroblast growth factor (FGF), vascular endothelial growth factor (VEGF), and connective tissue growth factor (CTGF), can promote fibrosis (Jiao et al., 2021; Zhang et al., 2021). The role of PDGF in fibrosis has been particularly well-studied. It has been shown that SiO_2_ can induce PDGF secretion in Tregs, thereby stimulating fibroblasts and promoting pulmonary fibrosis (Lo Re et al., 2011). The overexpression of PDGF aggravates silicosis by increasing Ca^2+^ release through the PI3K signaling pathway, ultimately influencing the expression of ECM-related genes in pulmonary fibroblasts (Mukherjee et al., 2013).

CTGF, the most direct downstream effector of TGF-β, influences the pathogenesis of silicosis by promoting the synthesis of ECM, the proliferation and migration of fibroblasts, and EMT (Paradis et al., 1999; Jiao et al., 2021). In silicosis, the TGF-β/CTGF pathway enhances inflammation and fibrosis primarily by interacting with Smad or MAPK (Jiao et al., 2021). A high concentration of CTGF is detected in fibroblasts treated with the supernatant of SiO_2_-stimulated AMs. However, anti-CTGF antibody administration prevents CTGF from activating downstream pro-fibrotic signaling, thereby attenuating silicosis (Cui et al., 2018). Combined, these observations indicate that CTGF exerts pro-fibrotic effects in silicosis.

VEGF plays a key role in lymphangiogenesis, which is an important channel for the removal of dust and inflammatory mediators in the early stage of silicosis (Yu et al., 2016). Jinsong et al. found that VEGF expression levels are higher in BALF and AMs of patients with silicosis than in healthy controls. In lymphatic endothelial cells cultured in supernatant medium of silica-treated human monocyte-macrophages (U937 cells), VEGF can promote the expression of VEGF receptor 3 (VEGFR3) and lymphatic vessel endothelial hyaluronic acid receptor 1 (LYVE-1), which can influence lymphangiogenesis by upregulating the Src/eNOS signaling pathway (Zhang et al., 2021). Specifically, VEGF stimulates lymphangiogenesis in silicosis, which promotes inflammation, oxidative damage, and, ultimately, fibrosis.

### 3.5 Chemokines

MCP-1 (also known as CCL2), mainly derived from fibroblasts and macrophages, is a member of the C-C class chemokine family and a key factor in the initiation of inflammation (Deshmane et al., 2009; Melgarejo et al., 2009). MCP-1 can regulate the migration, chemotaxis, and recruitment of macrophages and T lymphocytes during various types of inflammation (Lee and Song, 2017). Boitelle A et al. found that coal workers have higher levels of MCP-1 compared with healthy controls. They speculated that MCP-1 promotes the migration of peripheral blood mononuclear cells out of blood vessels into tissues, causing mononuclear macrophages to accumulate in the alveolar lumen, and thus influencing the occurrence of alveolitis in coal worker’s pneumoconiosis (Boitelle et al., 1997). When exposed to SiO_2_, human pulmonary fibroblasts (HPF-α) release MCP-1. The increased release of MCP-1 and its receptor, CCR2, promotes cell activation and migration (Liu et al., 2015).

Inflammatory factors play important roles in silicosis, the interaction between them is complicated. However, regulation of some of these factors suggest a strategy to mitigate the progression of fibrosis in silicosis, and some drugs targeting on these factors have revealed good therapeutic potential (Table 1).

**TABLE 1:** Drugs targeting inflammatory cytokines.

Name	Target	Mechanism	Ref
Anakinra	IL-1 receptor	↓collagen deposition	Cavalli et al. (2015)
		↓damaged lung	
		↓SiO_2_ nodule formation	
SM-17	IL-17 receptor B	↓the accumulation of neutrophils	Hasan et al. (2013)
		↓the development of Th17	
Anti-IL-17 Ab	IL-17	↑neutralization of IL-17	Chen et al. (2014)
		↓Th1/Th2 immune response	
		↓Th17 cells	
		↑Tregs	
Tralokinumab	IL-13	↑neutralization of IL-13	Murray et al. (2014)
		↓collagen deposition	
		↓myofibroblasts activation	
Infliximab	TNF-a	↓the expressing of NF-κB signaling	Zhang et al. (2018)
		↓M1 macrophage (iNOS)	
HTPEP-001	TGF-β	↓TGF-β reduction	Liu et al. (2023)
		↓fibrotic progress	
Pamrevlumab	CTGF	↓EMT	Cui et al. (2018)

## 4 Signal pathways

The inflammatory networks underlying the development of silicosis remain incompletely understood. Multiple pathways have been reported to participate in the pathology of silicosis.

### 4.1 The NF-κB signaling pathway

NF-κB is a nuclear transcription factor with a key role in pro-inflammatory signaling and represents a major regulatory node in a complex inflammatory signaling network (Lawrence, 2009; Rius-Pérez et al., 2020). NF-κB is rapidly and persistently activated in acute and chronic inflammatory disorders and plays a critical role in host defenses against pathogens and toxic substances, such as silica (Kang et al., 2000; Zhang et al., 2017; Dorrington and Fraser, 2019). Following exposure to silica, NF-κB is rapidly released from IκB, following which it translocates into the nucleus and initiates the transcription of specific genes, such as *TNF*, *IL1B*, and *IL6*, which, in turn, can further aggravate the inflammatory response and fibrosis (Rojanasakul et al., 1999; Porter et al., 2002). The systemic blockage of NF-κB activation can markedly ameliorate SiO_2_-induced inflammatory responses, collagen deposition, apoptosis, and fibrosis in mice. However, in transgenic mice with lung epithelial cell-specific inhibition of NF-κB activation, lung inflammation is decreased following silica exposure, whereas collagen deposition and apoptosis levels are increased (Di Giuseppe et al., 2009). As mentioned earlier, silica exposure induces NF-κB activation in macrophages, which modulates macrophage apoptosis. To clarify the role of NF-κB activation in silica-induced apoptosis, macrophages were treated with BAY 11-7085, a powerful inhibitor of IκB phosphorylation. The absence of NF-κB was found to increase silica-induced apoptosis in macrophages (Gambelli et al., 2004). The absence of NF-κB was found to increase silica-induced apoptosis in macrophages. Meanwhile, whereas the systemic inhibition of NF-κB protects against silica-induced lung injury, specific NF-κB inhibition in macrophages and lung epithelial cells appears to promote cell apoptosis and aggravate silicosis.

### 4.2 The MAPK signaling pathway

The generic MAPK signaling pathway is shared by four distinct cascades, namely, ERK1/2, JNK1/2/3, p38-MAPK, and ERK5. The MAPK signaling pathway is the primary regulatory module of various cellular processes, such as cell proliferation, differentiation, and responses to stress. ROS, cytokines, and other substances produced following exposure to SiO_2_ act on MAPK/ERK pathway-related receptors and activate downstream signals, thereby promoting silicosis formation and development (Ding et al., 1999). Studies have indicated that the SiO_2_-induced activation of fibroblast proliferation, ECM production, inflammatory responses, and apoptosis is mediated by the MAPK/ERK signal pathway, at least partly. The MAPK/ERK inhibitor (PD98059) exerts a marked antagonistic effect on silica-induced lung inflammation by inhibiting the expression of TNF-α and TGF-β (Li et al., 2009). Silica exposure induces ERK1/2 phosphorylation, which can protect against macrophage apoptosis, in contrast to that observed with PD98059 treatment (Gambelli et al., 2004). Silica induces early growth response protein 1 (EGR-1) activation via the MAPK/ERK pathway, resulting in the modulation of the expression of inflammation-related genes (PDGF, ICAM-1) and fibrosis-related genes that affect matrix balance (MMP1, fibronectin), which promotes the inflammatory response, fibroblast proliferation, and ECM synthesis (Zeng et al., 2005).

p38-MAPK, a central mediator of inflammatory and stress responses, is a major cellular signal transducer of extracellular stress signals induced by lipopolysaccharide (LPS), endotoxins, and pro-inflammatory cytokines (Yeung et al., 2018). p38-MAPK is activated by silica, following which it phosphorylates an array of substrates in both the cytoplasm and nucleus, thereby influencing inflammation, cell differentiation, and cell growth (Wang et al., 2016). Ample evidence suggests that p38-MAPK regulates signals involved in the development of fibrosis by mediating EMT and promoting TGF-β secretion. Treatment with SB203580, a p38 kinase inhibitor, can significantly reduce silica-induced TGF-β secretion and EMT, as evidenced by the consequent induction of E-cadherin and repression of vimentin and α-SMA. p38 inhibitor ameliorates silica-induced pulmonary fibrosis, which may be related to the inhibition of ZEB-1, ZEB-2, and Twist expression (Wang et al., 2016).

SiO_2_ is also known to activate JNK *in vivo and in vitro*. The JNK inhibitor BI-78D3 not only completely blocks the production of leukotriene B_4_, IL-1β, and CXC chemokines in macrophages, MCs, and neutrophils, but also reduces SiO_2_-induced sterile inflammation in an air-pouch model in mice (Hegde et al., 2018).

### 4.3 Fas/FasL

Pro-apoptotic Fas and FasL play critical roles in pulmonary immune homeostasis, immune surveillance, and autoimmunity involving T cells. Clinical research has shown that patients with silicosis exhibit elevated levels of soluble Fas in serum and peripheral blood mononuclear cells, as well as higher levels of other FAS transcript variants and reduced membrane Fas expression in lymphocytes (Otsuki et al., 2006). Fas/FasL signaling aggravates inflammation-related apoptosis in epithelial cells and AMs, which consequently release IL-1β and chemokines, leading to neutrophil infiltration and lung injury (Dosreis et al., 2004; Hoffmeyer et al., 2010). Patients with silicosis administered an anti-FasL antibody exhibit reduced levels of the pro-apoptotic factors Fas and caspase-3 and the inflammatory mediators TGF-β and IL-8 (Yao et al., 2013). Fas ligand-deficient GLD mice instilled with silica do not develop silicosis. These animals display significantly reduced neutrophil extravasation into the bronchoalveolar space, decreased TNF-α production, reduced pulmonary inflammation, and macrophage apoptosis (Borges et al., 2001). In wild-type mice administered an anti-FasL monoclonal antibody, neutrophil accumulation in the lung parenchyma is inhibited, as is macrophage apoptosis, and even silicotic fibrosis (Borges et al., 2001). The Fas/FasL pathway plays an important role in regulating the levels of inflammatory cytokines and promoting lung inflammation and fibrosis.

### 4.4 cGAS-STING

The cGAS-STING signaling pathway has emerged as an essential mediator of inflammation in infection, cellular stress, and tissue damage (Decout et al., 2021). Cyclic GMP-AMP synthase (cGAS) is a recently identified intracellular pattern recognition receptor (PRR) that can recognize abnormal cytoplasmic dsDNA, following which it catalyzes the synthesis of cyclic GMP-AMP (cGAMP). cGAMP then activates stimulator of interferon genes (STING), which promotes the secretion of type-I IFN and other inflammatory factors, thereby influencing the immune response (Motwani et al., 2019). The cGAS-STING pathway is essential for silica-induced lung inflammation. Activation of the STING pathway by airway silica leads to cell death, self-dsDNA release, an increase in ROS generation, and STING/type-I-IFN-dependent acute lung inflammation (Messaoud-Nacer et al., 2022). cGAS recognizes leaked dsDNA and activates STING *in vivo* after silica exposure. When DNase I is used to degrade dsDNA, silica-induced STING activation and the downstream type-I-IFN response are inhibited (Benmerzoug et al., 2018).

## 5 Inflammatory biomarkers

Owing to its hidden onset and long duration, silicosis is difficult to diagnose until severe lung injury occurs, at which point silicosis can be diagnosed based on an abnormal chest X-ray. However, at this stage, the lesions are irreversible (Jiang et al., 2015; Li et al., 2022). Accordingly, there is an urgent need to identify biomarkers for early silicosis diagnosis, i.e., before radiological alterations are detected. Several candidate biomarkers for silicosis have been identified, namely, the interleukins, TGF-β, TNF-α, GF, chemokines, inflammatory proteins, NLRP3, and DNA methylation. These biomarkers are mainly present in the cellular oxidative stress response, immune response, and tissue damage and repair processes, which play extremely important roles in the diagnosis of silicosis (Sato et al., 2006; Peeters et al., 2014; Perkins et al., 2016; Blanco-Perez et al., 2021).

### 5.1 Inflammatory cytokines

Ample evidence supports that SiO_2_ activates inflammatory cytokines such as IL-1β, IL-6, TGF-β, TNF-α, growth factors, and CCL and promotes pulmonary fibrosis (Boitelle et al., 1997; Blanco-Perez et al., 2021), suggesting that increased inflammatory cytokine levels may be used as typical biomarkers for the clinical diagnosis of silicosis.

### 5.2 Inflammatory proteins

HO-1, an inducible antioxidant stress protein, is one of the most important downstream regulatory effectors of nuclear factor-erythroid 2 related factor 2 (Nrf2) and exhibits anti-inflammatory and antioxidant properties (Loboda et al., 2016; Zhang et al., 2021). HO-1 is a rate-limiting enzyme in heme catabolism, degrading heme to carbon monoxide (CO), biliverdin (BV), and ferrous ion (Fe^2+^) (Ayer et al., 2016). It has been suggested that all these byproducts are important contributors to the anti-inflammatory activity of HO-1. The endogenous mediator CO inhibits the expression of pro-inflammatory cytokines, such as TNF-α and IL-1β, while simultaneously increasing the expression of the anti-inflammatory cytokine IL-10 (Abraham and Kappas, 2008). It is reported that the HO-1/CO axis regulates inflammation and the immune system (Ryter, 2020). BV and its downstream reductive derivative bilirubin (BR) are potent anti-inflammatory factors that can inhibit C5aR expression in macrophages and reduce pro-inflammatory cytokine expression through mTOR signaling (Bisht et al., 2014). HO-1 is present in silicotic nodules, where it exerts protective effects by attenuating lung inflammation (Nakashima et al., 2018), suppressing ROS activity and subsequent inflammatory and pathologic changes, and thereby attenuating disease progression (Sato et al., 2006; Nakashima et al., 2018). Low serum concentrations of HO-1 can predict impaired lung function in chronic silicosis. These observations imply that HO-1 is a key biomarker suitable for the monitoring of at-risk patients and a potential therapeutic target for silicosis (Sato et al., 2006; Sato et al., 2012).

The nuclear factor high-mobility group box 1 protein (HMGB1), which is actively secreted by inflammatory cells or passively released from necrotic cells, was recently found to function as an important late inflammatory mediator (Ciucci et al., 2011). High levels of HMGB1 have been strongly linked to silicosis; each 1 ng/mL increase in plasma HMGB1 levels is positively correlated with an increased risk of silicosis (Ma et al., 2018). Jixuan et al. demonstrated that the overexpression of HMGB1 induced by SiO_2_ exposure can influence the progression of silicosis, including pulmonary inflammation and fibrosis, via EMT.

The authors found that the neutralizing antibody-mediated abrogation of HMGB1 attenuated silica-induced lung inflammation, fibrosis, and EMT, whereas the application of recombinant HMGB1 exerted the opposite effect (Ma et al., 2020). HMGB1 acts to alert cells to hazardous surroundings, such as in the presence of silica. The increased secretion of HMGB1 can strongly promote leukocyte recruitment and activation, which triggers tissue repair and aggravates silicosis (Rabolli et al., 2014; Yang et al., 2020). Accordingly, there is sufficient evidence to show that HMGB-1 is involved in the progression of silicosis, suggesting that it may represent a potential diagnostic biomarker and therapeutic target for silicosis.

### 5.3 The NLRP3 inflammasome

The NLRP3 inflammasome is a large multi-protein complex composed of the core protein NLRP3, apoptosis-associated speck-like protein containing a CARD (caspase activation and recruitment domain) (ASC), and pro-caspase-1 (He et al., 2016). Once activated, caspase-1 cleaves inactive IL-1β and IL-18 into their mature forms, which generates pro-inflammatory effects (Jessop et al., 2017). Abundant evidence suggests that NLRP3-mediated inflammation plays an essential role in the fibrogenesis and pathogenesis of silicosis (Cassel et al., 2008; Peeters et al., 2014). NLRP3 inflammasomes in AMs are major inducers of cytokine secretion. The phagocytosis of SiO_2_ particles activates NLRP3 inflammasomes via four different mechanisms, namely: 1) SRs on the surface of AMs recognize and induce the internalization of SiO_2_ particles, leading to NLRP3 inflammasome activation (Hari et al., 2014); 2) AMs phagocytose SiO_2_, resulting in stress injury and the release of intracellular ATP to the outside of the cell. P2X7 receptors (P2X7R; ATP-gated ion channels) on the cell membrane recognize ATP, leading to the opening of some of the channels and the subsequent activation of NLRP3 by K^+^ outflow and Na^+^ influx (Luna-Gomes et al., 2015); 3) engulfed SiO_2_ particles promote the generation of a large amount of ROS, resulting in NLRP3 activation (Harijith et al., 2014); and 4), the contents released following lysosomal rupture activate the NLRP3 inflammasome. When activated, the NLRP3 inflammasome, *via* activated caspase-1, converts pro-IL-1β and pro-IL-8 to their active forms. IL-1β and IL-8 then act to increase the production of other pro-inflammatory and pro-fibrotic cytokines, which promotes fibrosis (Jessop et al., 2017).

Normal macrophages produce large amounts of IL-1β when exposed to silica crystals. However, ASC- and NLRP3-deficient mice both exhibit reduced inflammation and granuloma formation (Cassel et al., 2008). Macrophages lacking NLRP3 or ASC do not release cleaved IL-1β in response to silica crystal exposure (Hornung et al., 2008). Together, these findings indicate that the NLRP3 inflammasome plays a central regulatory role in silicosis, and further suggest that activated NLRP3 may serve as an important biomarker of silicosis.

### 5.4 DNA methylation

Dust inhalation can induce oxidative stress responses, leading to the generation of a large amount of ROS, reactive nitrogen species (RNS), lipid peroxides, and other free radicals, all of which can damage mitochondrial DNA and increase the incidence of DNA methylation (Fubini and Hubbard, 2003; Zhang et al., 2019). Aberrant DNA methylation profiles have been found in the lung tissues of patients with silicosis. Namely, 86,770 and 79,660 CpG sites were found to differ significantly in methylation status between the lungs of patients with early-stage and advanced-stage disease, respectively, and those of healthy individuals (Zhang et al., 2016). The DNA of a large number of genes is abnormally methylated in SiO_2_-treated lung fibroblasts. Methylated DNA immunoprecipitation (MeDIP) experiments showed that these genes are mainly involved in the transformation and differentiation of fibroblasts (Li et al., 2017). DNA methylation is also found in genes associated with the WNT signaling pathway, which contributes to proliferative, fibrogenic, and inflammatory responses to silica exposure in lung epithelial cells (Perkins et al., 2016). These findings suggest that specific DNA methylation may have value as a diagnostic biomarker for silicosis.

## 6 Treatment for silicosis

Although the pathogenesis and preventive treatment of silicosis have been explored for many years, the existing research on the mechanism of silica-induced pulmonary fibrosis is inadequate and lags far behind clinical needs. Lung lavage and transplantation are efficacious treatments available for patients with silicosis, however, both methods have some limitations (Pang et al., 2021). Many studies demonstrated that some pharmaceuticals are the promising drugs for the treatment of silicosis, which can address one or more target of silicosis to reduce silica-induced inflammation and/or fibrosis.

As mentioned earlier, inflammatory cells and cytokines are the most important factors in silicosis. Therefore, neutralizing cytokines and blocking cytokine receptors are the important mechanisms for the treatment of silicosis (Table 1).

In addition to acting specifically on the inflammatory system, other potential drugs have been reported to inhibit silica-induced pulmonary inflammation and fibrosis in silicotic models (Table 2). Pirfenidone and nintedanib are clinically used for treating idiopathic pulmonary fibrosis, which have been well documented in silica-induced fibrosis (Wollin et al., 2014; Wollin et al., 2015; Guo et al., 2019). Tetrandrine, approved for silicosis in China, has been demonstrated a value regent in clinic therapy for many years (Bhagya and Chandrashekar, 2016). Some researches demonstrated the oxidative stress encountered during pulmonary fibrosis is closely associated with inflammation. N-acetylcysteine (NAC) and tanshinone IIA have been found to be beneficial in the management of silicosis (Zhang et al., 2013; Zhu et al., 2016). Other drugs also show great therapeutic potential in silicosis, such as drugs affecting the autophagy-apoptosis system (dioscin, trehalose, and atractylenolide III), drugs inhibiting macrophage polarization (bicyclol), drugs inhibiting inflammatory cell (TY-51469), and drugs degrading the activity of NLRP3 inflammasome (tetrandrine, MCC950).

**TABLE 2:** Drug candidates for the treatment of silicosis.

Name	Mechanism	Ref
Nintedanib	↓block FGF receptor-1	Wollin et al. (2014), Wollin et al. (2015)
	↓block PDGF receptor	
	↓*Src* pathway	
Pirfenidone	↓macrophage polarization	Li et al. (2018), Guo et al. (2019), Cao et al. (2022), Tang et al. (2022)
	↓IL-17A	
	↓TAK1-MAPK-Snail/NF-κB pathway	
Tetrandrine	↓NLRP3 activation	Bhagya and Chandrashekar (2016), Song et al. (2022)
N-acetylcysteine	↓NF-κB activation	Zhang et al. (2013)
Tanshinone IIA	↓inflammatory cells (neutrophils, macrophages and lymphocytes)	Zhu et al. (2016)
	↓TNF-α, IL-6, IL-1β	
Dioscin	↑AMs autophagy	Du et al. (2019)
	↓AMs ROS	
	↓AMs apoptosis	
	↓secretion of inflammatory factors and chemokines.	
Trehalose	↑AMs autophagy	Tan et al. (2020)
	↓AMs apoptosis	
Atractylenolide III	↓AMs autophagy by mTOR-dependent manner	Chen et al. (2021)
	↓AMs apoptosis	
	↑blockage of autophagic degradation in AMs	
Bicyclol	↓macrophage polarization	Zhan et al. (2021)
TY-51469	↓chymase in mast cells	Takato et al. (2011)
	↓neutrophils	
MCC950	↓NLRP3 activation	Lam et al. (2022)
	↓IL-1β, IL-18	

In conclusion, an excessive immune response caused by overactive adaptive and innate immune cells and their cytokines is the primary pathogenic mechanism underlying the development of silicosis, leading to immune imbalance and collagen deposition.

Despite the large number of studies that have investigated the role of inflammation in fibrogenesis, there is insufficient evidence relating to how the inflammatory networks regulate fibrogenesis. Inhaled SiO_2_ particles induce the recruitment of neutrophils and macrophages to the lungs, the production of pro-inflammatory mediators, cell death, and fibroblast activation. The incomplete clearance of SiO_2_ results in chronic inflammation, the formation of silicotic nodules, fibrosis, and impaired pulmonary function. Many immune molecules are involved in this process. However, the intricate interrelationships and molecular mechanisms among the various immune cells, inflammatory proteins, and inflammatory-related pathways remain poorly understood. No specific anti-inflammatory drugs for the treatment of silicosis are currently available. However, clinical trials like anti-IL-13 and anti-IL-17 antibodies are presently underway. With a better understanding of the pathogenesis of silicosis, an increasing number of possible therapeutic targets and underlying mechanisms have been identified, and drugs with therapeutic potential have been and continue to be developed.

## Figures and Tables

**Table:** 

**AMs**	Alveolar macrophages
**ASC**	Apoptosis-associated speck-like protein containing a CARD
**APCs**	Antigen-presenting cells
**BCR**	B-cell receptor
**bFGF**	Basic fibroblast growth factor
**BR**	Bilirubin
**Bregs**	Regulatory B cells
**BV**	Biliverdin
**CARD**	Caspase activation and recruitment domain
**cGAMP**	Cyclic GMP-AMP
**cGAS**	Cyclic GMP-AMP synthase
**CO**	Carbon monoxide
**CTGF**	Connective tissue growth factor
**DCs**	Dendritic cells
**ECM**	Extracellular matrix
**EGR-1**	Early growth response protein 1
**HMGB1**	High-mobility group box 1 protein
**FGF**	fibroblast growth factor
**IL**	Interleukin
**iNOS**	inducible nitric oxide synthase
**LPS**	Lipopolysaccharide
**MCs**	Mast cells
**MeDIP**	Methylated DNA immunoprecipitation
**MMPs**	Matrix metalloproteinases
**NE**	Neutrophil elastase
**NETs**	Neutrophil extracellular traps\
**NEUTs**	Neutrophils
**NLRP3**	NOD-like receptor thermal protein domain associated protein 3
**Nrf2**	Nuclear factor-erythroid 2 related factor 2
**PAR2**	Proteinase-activated receptor 2
**PDGF**	Platelet derived growth factor
**PRR**	Pattern recognition receptor
**RNS**	Reactive nitrogen species
**RORγt**	Retinoid-related orphan nuclear receptor γt
**ROS**	Reactive oxygen species
**SiO**_**2**_	Silicon dioxide
**SRs**	Scavenger receptors
**STING**	Stimulator of interferon genes
**TCR**	T-cell receptor
**TGF-β**	Transforming growth factor β
**Th**	T-helper
**TIMP**	Tissue inhibitor of metal protease
**TNF-α**	Tumor necrosis factor α
**Tregs**	Regulatory T cells
**TβR**	TGF-β receptors
**VEGF**	Vascular endothelial growth factor
